# Starting from the future by looking to the past: reclaiming radical pedagogies, charting cartographies of imagination

**DOI:** 10.3389/fmed.2026.1889513

**Published:** 2026-08-25

**Authors:** Nivethitha Ram Ganapathiram, Andy Guise

**Affiliations:** Faculty of Life Sciences & Medicine, King's College London, London, United Kingdom

**Keywords:** anti-discrimination, critical pedagogy (CP), epistemology - education, medical education, social justice

## Abstract

This perspective piece offers a theoretical and pedagogical framework for delivering meaningful social justice education in medical curricula, grounded in the design of an anti-discrimination pilot programme for undergraduate medical students. Drawing on John Dewey, Paulo Freire, and bell hooks, we examine three contemporary challenges—neoliberal pedagogies, technological determinism, and manufactured scarcity—and propose three entangled responses: relational community-building, conscientização, and vulnerability. Playing with the linearity of time, itself a colonial logic, we reach back to enduring knowledge in tandem with working backwards from imagined futures, drawing our cartography of imagination.

## Introduction

“*We shed as we pick up, like travellers who must carry everything in their arms, and what we let fall will be picked up by those behind. The procession is very long, and life is very short. We die on the march. But there is nothing outside the march so nothing can be lost to it.” — Stoppard* *(**1**)*

The notion of “social justice” is complex and contextual; therefore, it is important to describe what it means to us, before operationalizing it in our curricula. We understand social justice in health through the lens of Venkatapuram's (2) capability approach, in which health is not simply a biomedical state, but the freedom to access the social, political, and material conditions that make a healthy, meaningful life possible. This extended understanding of health imagines an ambitious vision for medical education, calling for it to cultivate clinicians who can engage *structurally* with their patients (3).

This structural engagement reaches beyond clinical competence, into the cultivation of what McKenna (4) calls “constructive troublemakers.” That is, clinicians who have been trained to see the socio-political machinery that underlies pathology, and to act on what they find. This kind of training seeks to unpick bit by bit the coloniality that has been knitted into the epistemology of medicine. We understand coloniality here as a totalizing logic of *power*, organizing whose knowledge is valid and whose suffering is visible (5).

Departing from this understanding of social justice education, this piece seeks to set out a theoretical and pedagogical framework for medical curricula, grounded in the practical design of an anti-discrimination pilot programme for undergraduate medical students, from which these ideas have iteratively emerged. Playing with the linearity of time, itself a colonial logic, we reach back to enduring knowledge in tandem with working backwards from imagined futures. In the ink of radical constructivist pedagogy (through Dewey), Latin American decolonial theory (through Freire), and Black feminist theory (through hooks), we draw our cartography of imagination. Maps, as Young and Varpio (6) remind us, are epistemic choices that tell us particular stories. A cartography of imagination is an act of counter-narration that writes into being an alternative medical curriculum; one which makes different epistemic choices and therefore tells new, different stories. These three educators, writing across three distinct historical moments, all forged their ideas in and against conditions of hegemonic power to imagine a counterfactual world. To start from the future is to imagine the counterfactual world and, from *there*, work backwards to construct the theory and praxis that breathe it into life.

The ground was moving beneath the feet of all three educators, and we find ourselves in a socio-political moment of no less seismic instability: neoliberal logics reduce education (and health) into commodities, fracturing collective resources for individual transaction; artificial intelligence encodes algorithmic bias into the datasets that train our future clinicians; and capitalist logics of scarcity deplete the time, joy, and vulnerability on which genuine professional formation depends. The ground is moving beneath our feet too; and so, we are called to pick up these radical ideas, re-imagine them to meet the challenges in our contemporary practice, and carry them into the future we are building; not despite the instability, but because of it.

For us, this means accepting that the tools and conditions we need to deliver medical curricula rooted in community, critical consciousness, and vulnerability, do not yet fully exist; they must be imagined into being. Imagining across a non-linear axis of time is where critical awareness converges with critical vision, and it is the precondition for transformative action. This work of *imagining* is not an academic indulgence to be set in opposition to the gritty work of *doing*. Imagining the epistemic architecture is what guards practice against defaulting into what Mignolo (7) calls “decolonial washing”: the institutional adoption of social justice language that leaves the invisibilized colonial matrix of power entirely undisturbed. As Manzoor-Khan [(8), p. 81] writes in her poem *De-centring Diversity*: “More people of color on pedagogically unchanged reading lists is not salvation.”

In this piece, we examine three challenges we have encountered in our contemporary practice: neoliberal pedagogies, technological determinism, and manufactured scarcity. Our analysis is refracted through the lens of Dewey's theory of school as community life, Freire's theory of critical consciousness, and hooks' theory of engaged pedagogy. These theories also inform the design of our anti-discrimination pilot programme. We define the programme's aims relationally, not by competency; we co-produce its curriculum with students; and we centre art and narrative as epistemic resources within it. Recognizing that our specific design choices are not neatly transferable conclusions, we close each section with questions of departure as invitations for educators to reflect on their own practice, in their own context, in their own time.

## Dewey: community in the age of neoliberal pedagogies

“*…the best and deepest moral training is precisely that which one gets through having to enter into proper relations with others in unity of work and thought.” [(**9**), p. 8]*

Contemporary medical curricula are structured with a near totalizing commitment to measuring outcomes and “performing” competence through psychometric assessment and audit culture (10). This has emerged within a wider neoliberal rationality across education that conceptualizes learning as a metric of competition, governance, and commodification (11). In medical education the neoliberal logic gained traction as a *technical* response to the very real problem of inconsistent standards and unchecked gatekeeping; however, its cascading *epistemic* consequences are laid bare in social justice education, or more precisely, the current limits on social justice education. When we teach on social justice within a medical curriculum we encounter a pedagogical friction arising from an emphasis on outcomes.

We begin to imagine a future where *relational* pedagogies are the architecture of our medical curricula by reaching back to Dewey's radical theory of the school as “*that form of community life”* where we “*share in the inherited resources”* to use our “*own powers for social ends.”* [(9), p. 6].

Thinking in the late 19th century at the height of American industrialization, when rapid technological change demanded that education reduce itself to the production of compliant, competent workers (who in turn produced commodified goods), Dewey (9) re-imagined education as a collective, experiential practice that draws on dialogic and relational resources to produce and sustain the common good.

Experiential learning models, such as Kolb's (12) cycle and Mezirow's (13) transformative learning, deeply encode praxis within contemporary medical curricula. Though they grew from Deweyan roots, in their uptake, we see them progressively detached from their politically engaged origins: with reflection relocated within an individual's cognition, and re-oriented toward attaining individual competence or transformation. We reclaim the Deweyan trans-actional approach which conceives education as a cooperative, reflexive act that dynamically transforms the individual and the system iteratively and in parallel (14).

For Dewey (9), learning is constituted by the quality of the relationships within which it happens, because knowledge is dynamic, emergent, and produced through dialogue and shared inquiry. Meaningful social justice education lives and grows in these relational dimensions of learning that, in their richness and expansiveness, elude measurement and audit and are often undermined by it. It finds itself contorted in neoliberal pedagogies, wherein knowledge is divorced from people and deformed into individual capital that flows through regimes of performativity (11). We imagine an alternative to this by employing Dewey's relational pedagogy as the organizing principle of our anti-discrimination pilot programme. We start by considering what building trust and belonging within pedagogical relationships practically requires.

First, we define the aims of the programme not in the metrics of competency (what learners can do), but in the relational process of professional formation (who learners and facilitators are becoming). The purpose of the programme is not to transmit social justice as pre-determined knowledge, but to embody it as an emergent process of building community and belonging. Our role as educators is not to map out objectives, but to map out the practices and conditions that will support and scaffold these processes, the ends of which will be plurally experienced, and so cannot be uniformly measured.

In our programme, teaching and learning are not discrete activities but stirred into each other. This is what Vygotsky called *obuchenie*: the same word for teaching and learning; an active collaboration between student and teacher through which both become (15). In practice, this means closing a session not with “*what is one thing you have learned today?*” but with “*what did relating to each other look like today? what worked, and what didn't”*—orienting inquiry not toward the knowledge that emerged from relating, but toward the quality of the relating itself, and how we might be more fully present with and for ourselves and each other.

Second, these aims require structural scaffolding: regularity, continuity, and familiarity. In practice, this means five fortnightly sessions across a term, with the same small group of six students and the same two facilitators (the co-authors) throughout. This scaffolding is the fascia of our relational pedagogy; without it, it is disembodied. This design also responds to empirical evidence demonstrating that fragmentation of clinical training through block rotations actively erodes empathy and patient-centredness over time, while continuity of relationship not only prevents this erosion but creates the conditions for the constructive, action-oriented exchange through which genuine professional formation occurs (16–18). Box 1 offers questions of departure to invite readers to reflect on Dewey's theories as they relate to their own teaching practice.

Box 1 **Questions of departure: Dewey.**

• How does your teaching practice actively build trust with your students? What choices in the design and delivery of your teaching signal to students that they belong?

• Do you feel in community with your students, and do they in turn feel in community with you?

• What might you do differently in your next teaching session to decentre performance and outcome, and move toward a collective process of inquiry?

## Freire: *Conscientização* in the age of technological determinism

“*To begin always anew, to make, to reconstruct, and to not spoil, to refuse to bureaucratize the mind, to understand and to live life as a process—live to become…” [Freire, cited in* *(**27**), epigraph]*

As we defined at the outset, social justice education trains clinicians to see the socio-political machinery that underlies pathology. Put another way, it trains them to see what they have been pedagogically conditioned to unsee. Therefore, this kind of education must begin with *unlearning*. We understand unlearning as Ortaçtepe Hart (19) reconceptualizes it: not as an individualized process solely directed at introspective self-reflection; but as a politicized, collective process directed at structural reflexivity and action.

In the current context in which Large Language Models (LLMs) permeate every inch of our medical curricula, the longstanding disillusionment with audited reflection finds its release in the automation of it. One study found that ChatGPT can produce high-quality written reflections that “outperform” student writing in assessment criteria, with human evaluators unable to accurately differentiate between AI-generated work and original student work (20).

There is mounting concern that LLMs bypass the productive struggle through which deep, collective (un)learning occurs, short-circuiting reflexivity and atrophying criticality and creativity (21). Pedagogical responses to this have been laced with technological determinism: the process by which technology autonomously re-structures the architecture of our social, political, and cultural worlds (22). Broadly, this looks like the uncritical adoption of LLMs into curriculum design (an “if you can't beat them, join them” strategy); or the individualization of blame onto students by means of surveillance and penalty. While there is growing literature that addresses AI and reflective practice in medical education directly, it focuses on toolkits, competencies, and individual digital literacy, even where it gestures toward the alienation we describe (23, 24). Such responses can individualize the problem rather than reckoning with the structural conditions that produce it. They do not stand up when designing pedagogies of unlearning necessary for social justice education. We therefore reach back to Freire's (25) anti-colonial theory of *conscientização* to imagine a counterfactual to this technological determinism.

Educating under the shadow of military dictatorship and systemic colonial violence in post-coup Brazil, Paulo Freire conceptualizes the role of an educator as someone who cultivates curiosity and critical inquiry to bring about liberation, personally, and politically (25). Conscientização is the process by which we develop a critical consciousness of the structural forces that shape our world, and the collective capacity to transform them (25). It emerges at the convergence of reflection, dialogue, and praxis. The automation of reflection is not merely a problem but an onto-political one: a “regime of neutralisation” that disarms the very subjective capacities for collective political imagination and action that conscientização seeks to cultivate (22).

In our anti-discrimination programme, we root unlearning in the ritual of conscientização through a design of co-production. Co-production is a way of redistributing epistemic power that is well established in healthcare research but seldom applied to curriculum design and delivery (26). We will start sessions with questions of departure (much like in this article) rather than learning objectives, priming the space for curiosity and dialogue, rather than unilateral transmission. Opening with questions positions us as co-enquirers, rather than as experts, creating the conditions for relational equity. The curriculum emerges in response to these questions, inviting the students to bring their narratives of experiencing, witnessing or, at times, reproducing discrimination in clinical practice. The questions offer, rather than prescribe, a shared hermeneutic resource—we hold them as threads to feel our way along together, rather than shackles that restrict how far we can go.

The specificity of lived experience sieved through the collectivity of dialogue produces a thick, local constellation of knowledge that cannot be algorithmically predicted. This co-creation exercises our reflexivity and relationality, producing responses that imagine otherwise—ones that students carry into practice and that reshape the programme itself. The learning moves in every direction and occurs at the convergence of reflection, dialogue, and praxis. Carving out time and space for this process to unfurl is our resistance to technological determinism, and our reclaiming of epistemic agency. It is a pedagogical choice that recognizes that students reach for automation out of alienation and precarity, and responds not with surveillance, but with nurture. Box 2 offers questions of departure to invite readers to reflect on Freire's theories as they relate to their own teaching practice.

Box 2 **Questions of departure: Freire.**

• What would you like to unlearn as a teacher/learner? What would this unlearning look like as collective process directed at action?

• How could you re-distribute epistemic power in your teaching design and delivery? What does this look like in practice?

• What is obstructing conscientização in your practice? What do you need to nurture it?

## hooks: joy in the age of scarcity

“*School was the place of ecstasy…To be changed by ideas was pure pleasure.” [(**27**), p. 3]*

Over a century ago, when Rudolf Virchow contended “medicine is a social science and politics is nothing else but medicine on a large scale,” he located the clinician within the community, as much as the clinic (4). And yet, though the case for social justice education within medicine is longstanding, its integration remains uneven (28). Time and resource scarcity in overcrowded, balkanized curricula are often cited as the key barrier. This is reflected in our own practice, where the curriculum has often demanded that big ideas are squeezed into fractured, atomized moments, and slow work is accelerated.

We understand this as capitalist logic structurally manufacturing scarcity, conceptualized by Rosa (29) as a “contraction of the present.” The outpacing of our affective, cognitive, and bodily capacities by rapid techno-social acceleration shrinks the time and space in which personal and collective transformation is possible (29). In this temporal architecture, he argues that “standing still is equivalent to falling behind,” [(29), p. 11].

Having internalized this logic, medical education renders the slow, relational work of social justice pedagogy structurally incommensurable with the curriculum's own logic of efficiency.

When high-stakes, “objective” psychometric assessment becomes the organizing principle of ever-expanding competency frameworks, we design curricula that impoverish students' time and capacity to learn relationally and affectively—with their teachers, their peers, and their patients. That is, we diminish their capacity to *feel* the “pure pleasure” of being changed by ideas [(27), p. 3].

Educating from within the deep racial polarization of the late 20th century academy in the United States, hooks writes the Black feminist canon into critical pedagogy by re-centring the affective and the embodied. For hooks (27), education is a practice of freedom in which feeling and thinking are inextricable; where excitement, joy, and vulnerability are not confounders of rigorous inquiry, but it's very conditions (27). These feelings are disruptive to dominant epistemes and temporalities because they demand our openness to risk: flexible agendas, spontaneous trajectories, and plural knowledge. They call for explorative pedagogies kneaded in joy and vulnerability to resist the currency of utility and efficiency.

In our programme, we design what we might call a *felt curriculum*, that draws on academic work but also on art, music, literature, spoken word, and our own personal narratives as epistemic resources. Building community across asymmetries of power requires vulnerability as an epistemic practice, and a safe way of doing that in a classroom is through art. Art compels feeling, allows play, and transpires communion (30, 31), shirking off what McKenna (4) cites as the “banality of reason”: the mechanism by which empiricism depoliticizes, teaching structural violence taxonomically rather than viscerally. We offer this not as a fixed list, but as a living resource, to which everyone in the room (learners and facilitators) can contribute. For example, rather than beginning a session on the structural determinants of health with epidemiological data, we begin with Lasoye's (32) *Manifesto (see* *Box 3**)*: a poem that compels us to confront housing, migration, policing as felt realities, rather than abstracted data points, before asking of us: what version of ourselves do we need to leave behind for “the new world” to be possible?

Box 3 **Excerpt from *Manifesto (Lasoye, 2024)*.**

No eviction here. No rent.

No real estate agents. No real estate.

Land back. Settlers dealt with.

Nothing will be missed.

Not the old maps or the high rises.

Nothing will be missed.

Not private gardens.

Not fanciful lines drawn in the air.

Not any kind of border — especially not the ones in our heads,

the psychic invention of a divide between You and I.

Nothing will be missed.

Not people yelling *the time is now!* to people for whom this is not their first apocalypse.

(If this is your first crisis, welcome. If this is your first crisis, no, it's not.)

Nothing will be missed.

Not compromise, not capitulation, not widening participation, not incrementalism.

Not *forty per cent reduction in blah blah by twenty-whatever*.

Nothing will be missed.

Not Global Health Governance.

Not International Development.

Not the stabilisation of global power imbalances

under the guise of challenging global power imbalances.

Nothing will be missed.

Building conscientização across asymmetries of power requires an engaged pedagogy (25, 27). In our programme, we give back what we ask for: asking participants to write a reflective account at the close of the programme and doing the same ourselves. We share these reflections with them, inviting their critique and challenge, just as we offer ours of theirs. We fold vulnerability bilaterally into the assessment as its embodied companion, recognizing analysis itself as a form of power. Willis (33) argued some three decades ago that curricular reform without assessment reform will not yield deep learning. We extend this further: the reflective account is not merely an assessment, but a co-creation that embodies the epistemic commitments of our felt curriculum—that is, the relational, the critical and the affective domains of social justice education. Box 4 offers questions of departure to invite readers to reflect on hooks' theories as they relate to their own teaching practice.

Box 4 **Questions of departure: hooks.**

• Does your teaching practice make space for feeling alongside thinking? What could you build into your teaching to make joy and excitement possible?

• What epistemic resources is your teaching currently built on? Is there an opportunity to bring art and narrative (yours, the learner's or that of patients') into your curriculum?

• Is there space for vulnerability in your teaching? What practice or ritual could build that space, and what would it mean to give back what you ask of your students?

## Positionality and reflexivity: transgressing with our *beloved community*

This piece, and the teaching it imagines, has emerged from a longstanding mentorship between the two co-authors. We too relate across asymmetries of power, refracted through age, race, gender, seniority, and institutional capital. Reflecting on this, we see how we have imagined community, conscientização and vulnerability through embodying them from within our relational practice. The *how* of this is not neatly mappable onto a toolkit or framework, because it is a *felt* sense of accompliceship (19). There is laterality of stakes and a redistribution of risk, even if the specificities of what this means look different for each of us. Equally, these are not abstract commitments, and they can be traced to *felt* moments, specific to our contexts, needs, and capacities. For example, AG offering up his “half-baked” work for NG's comment, created the relational conditions for her to feel brave to do the same. There are many more quiet practices that have ritualized theory, making it felt and alive, not merely intellectualized and inert.

## Conclusion: charting the cartography of the imagination

Social justice education is fundamentally something we do *with* others, not *to* others; both the means and the ends are necessarily collective. Drawing on three radical educators, our own contemporary practice and our future aspirations for medical education, we chart a cartography of imagination across time, not sequentially, but iteratively. What emerges are entangled responses from educators across different times and contexts who constitute a larger whole; accomplices with each other (and with us) in co-creating relational pedagogies of unlearning and felt curricula. We have mapped this entangled architecture in Table 1.

**Table 1:** Mapping contemporary challenges, theoretical frameworks, and programme design across Dewey, Freire, and hooks.

Dimension	Dewey: relational pedagogies	Freire: pedagogies of unlearning	hooks: the felt curriculum
**Contemporary Challenge**	**Neoliberal pedagogies:** audit culture and psychometric assessment reduce learning to a metric of competition, governance and commodification	**Technological determinism:** automated reflection bypasses the productive struggle through which critical consciousness is collectively built	**Manufactured scarcity:** overcrowded, balkanized curricula render the slow, relational work of social justice education structurally incommensurable with the curriculum's logic of efficiency
**Theoretical framework**	• Learning as form of community life • Knowledge as collectively constructed • Education as the reconstruction of experience	• Learning as the development of critical consciousness • Knowledge as inseparable from one's social and political reality • Education as building collective capacity to transform structural forces of oppression	• Learning as affective and embodied • Knowledge as plural and emergent • Education as engaged pedagogy rooted in joy and vulnerability
**Programme Design**	• Aims defined relationally, not by competency • Structural scaffolding: regularity, continuity, and familiarity	• Co-production of sessions • Beginning with questions, not objectives • Lived narratives as core curriculum content	• Co-creation of felt curriculum; art as an epistemic resource • Reciprocal reflective account • Bilateral vulnerability folded into assessment
**Entangled responses**	Community-building 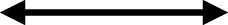	Conscientização 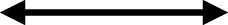	Vulnerability

Dewey (9) helps us imagine the relational conditions social justice education needs to take root; Freire (25) then helps us imagine how we name and interrogate power within those relationalities; hooks (27) then helps us imagine how we embody vulnerability across those asymmetries of power we have named. We see our place in the procession, looking back and ahead in tandem, and understand our work as part of intergenerational solidarities. This disrupts colonial temporalities at work within dominant medical pedagogy, and in doing so, expands our capacity to reflect, to relate and to imagine. An imagination we stand still inside, hand-in-hand, long enough to be changed.

## Data Availability

The original contributions presented in the study are included in the article/supplementary material, further inquiries can be directed to the corresponding author.
